# A simulation model of *Escherichia coli *osmoregulatory switch using E-CELL system

**DOI:** 10.1186/1471-2180-4-44

**Published:** 2004-11-30

**Authors:** KV Srividhya, Sankaran Krishnaswamy

**Affiliations:** 1 Bioinformatics Centre, School of Biotechnology, Madurai Kamaraj University, Madurai 625 021, Tamil Nadu India

## Abstract

**Background:**

Bacterial signal transduction mechanism referred to as a "two component regulatory systems" contributes to the overall adaptability of the bacteria by regulating the gene expression. Osmoregulation is one of the well-studied two component regulatory systems comprising of the sensor, EnvZ and the cognate response regulator, OmpR, which together control the expression of OmpC and OmpF porins in response to the osmolyte concentration.

**Results:**

A quantitative model of the osmoregulatory switch operative in *Escherichia coli *was constructed by integrating the enzyme rate equations using E-CELL system. Using the substance reactor logic of the E-CELL system, a total of 28 reactions were defined from the injection of osmolyte till the regulated expression of porins by employing the experimental kinetic constants as reported in literature. In the case of low osmolarity, steady state production of OmpF and repression of OmpC was significant. In this model we show that the steady state – production of OmpF is dramatically reduced in the high osmolarity medium. The rate of OmpC production increased after sucrose addition, which is comparable with literature results. The relative porin production seems to be unaltered with changes in cell volume changes, ATP, EnvZ and OmpR at low and high osmolarity conditions. But the reach of saturation was rapid at high and low osmolarity with altered levels of the above components.

**Conclusions:**

The E-CELL system allows us to perform virtual experiments on the bacterial osmoregulation model. This model does not take into account interaction with other networks in the cell. It suggests that the regulation of OmpF and OmpC is a direct consequence of the level of OmpRP in the cell and is dependent on the way in which OmpRP interacts with *ompF *and *ompC *regulatory regions. The preliminary simulation experiment indicates that both reaching steady state expression and saturation is delayed in the case of OmpC compared to OmpF. Experimental analysis will help improve the model. The model captures the basic features of the generally accepted view of EnvZ-OmpR signaling and is a reasonable starting point for building sophisticated models and explaining quantitative features of the system.

## Background

Among prokaryotes, a remarkable number of cellular functions are controlled by two component regulatory systems [1]. Dedicated circuits transduce and interpret specific signals such as pH, temperature, osmolarity, light, nutrients, ions, pheromones and toxins to regulate a wide range of processes including motility, virulence, metabolism, the cell cycle, development switches, antibiotic resistance and stress responses in a variety of systems from prokaryotes, archaea and eukaryotes [2].

Two component systems interact with each other and also with other regulatory systems mediating specific gene expression or cellular locomotion. Such complexes should be analysed quantitatively. Although quantitative data on cellular processes other than metabolism are still relatively sparse, scientists have modeled some systems with considerable success [3]. For example computer simulations of the chemotaxis two component system have been extensively studied [4].

**Figure 1:**
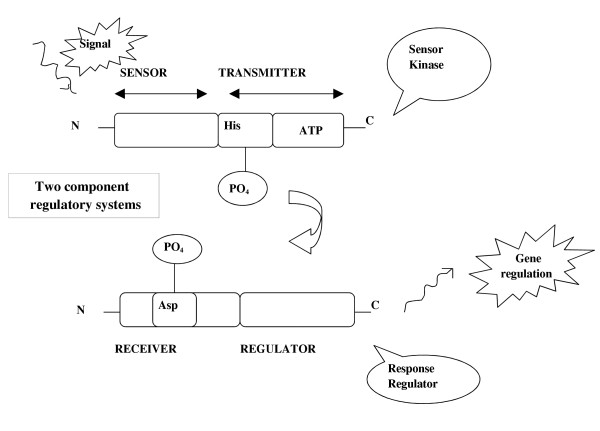
Two Component Regulatory Systems. The first component, sensor kinase autophosphorylates and transfers phosphate to the response regulator. These are also called the HAP systems indicating the involvement of Histidine of sensor kinase and Aspartate of Response regulators playing a key role in signal transduction.

Two-component regulatory systems (also called the HAP or His-Asp phosphotransfer mechanism) are widely used signaling machinery of bacterial adaptive responses. The system constitutes of sensor kinases and response regulators that can be phosphorylated and dephosphorylated by sensor kinases [5] (Figure 1).

The first component sensor-transmitter that spans the cytoplasmic membrane has two domains a sensory domain and a transmitter domain. The two domains are anchored in the cytoplasmic membrane by two membrane-spanning regions. The transmitter domain is capable of autophosphorylation utilizing ATP. During this reaction a phosphate group (PO_4_) is transferred to a specific Histidine (His) residue in the protein. The transmitter domain possesses a kinase activity that enables it to transfer the phosphate group to the receiver-regulator, when the environmental signal is detected [6]. The receiver-regulator that is cytoplasmically localized also consists of two domains. The first domain has a specific Aspartate (Asp) residue that can accept the phosphate group (PO_4_) from the transmitter domain. The second domain is the regulator that in response to phosphorylation can bind to specific DNA sequences near specific promoters to activate gene expression. These two domains are linked together by a flexible linker [7].

Osmoregulation is one of the well-studied two component regulatory systems operative in *Escherichia coli*, playing an important role in regulating the cellular response to different solute concentrations in their environment [8]. Among such types of osmotic responses, the expression of the major outer membrane proteins, OmpC and OmpF, has been the subject of extensive studies [9]. Both the OmpC and OmpF proteins form passive diffusion pores in the outer membrane, which facilitates the diffusion of small hydrophilic molecules across the membrane [10]. EnvZ the sensor kinase serves as a substrate for the phospho-transfer to aspartate-55 of OmpR, the response regulator [11,12]. EnvZ is a bifunctional histidine kinase that exhibits dual opposing functions, both phosphorylation and dephosphorylation of OmpR [13,14].

The sensor kinase is triggered by the osmolyte concentration of the environment and controls the production of phosphorylated regulator OmpR. This leads to the expression of outer membrane proteins OmpC or OmpF [15]. Phosphorylated OmpR binds to the regulatory sequences upstream of the *ompC *and *ompF *promoters [16,17]. OmpR undergoes a conformational change based on phosphorylation [18] and regulates the expression of the porin genes *ompF *and *ompC *in *Escherichia coli *[19]. The osmolyte works as the initial control element of the phosphorylation cascade. The primary signal for such a conformational change may be that caused by a change in the physical membrane-tension due to osmotic pressure [20]. First, the EnvZ-dimer, in the cytoplasmic membrane senses the environmental osmotic stimulus. The N-terminal membrane-spanning and periplasmic domains of the EnvZ-dimer presumably can take on two alternative conformational states (*i.e*., a high osmolarity form and a low osmolarity form) regulated by the osmotic signal and modulates the ratio of the kinase to phosphatase activity of EnvZ [21,22]. Under low osmolyte concentrations, Phosphatase activity of EnvZ predominates the kinase activity resulting in the binding of phosphorylated OmpR to the high affinity promoter of *ompF *gene triggering OmpF expression. In case of high osmolyte concentration the kinase activity of EnvZ is triggered resulting in the binding of the phosphorylated regulator to the low affinity promoters of *ompC *gene favouring OmpC expression [23].

## Events associated with high osmolarity

**Figure 2:**
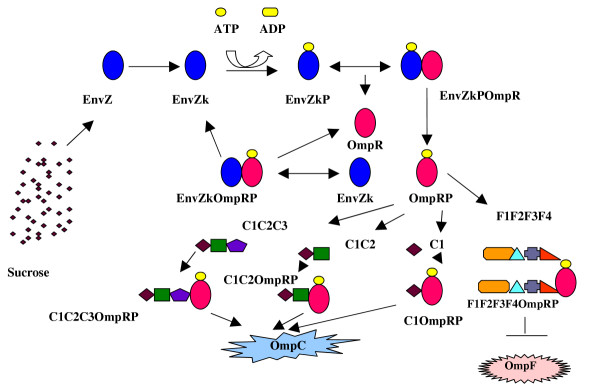
**Molecular Model of Osmoregulatory switch operative at High osmolarity in *Escherichia coli***. With the injection stimulus of sucrose as osmolyte the sensor EnvZ is triggered to take up either kinase or phosphatase activity. In high osmolarity conditions the higher osmolyte medium (NB (Nutrient Broth)+20% sucrose) makes the kinase activity of EnvZ (EnvZk) predominate the phosphatase activity resulting in the formation of EnvZkpOmpR complex. Finally after the phosphotransfer of phosphate group to OmpR, the promoter sites of OmpF are occupied in cooperative manner with F1, F1F2 and F1F2F3, high affinity binding sites. The cellular concentration of OmpRP makes it available for the OmpC promoter C1, C1C2, C1C2C3. Additionally OmpRP binds to F4 after binding F1F2F3 promoter directly repressing OmpF expression, thereby facilitating OmpC expression.

In the high osmolarity state, EnvZ actively undergoes autophosphorylation at histidine residue-243 in the C-terminal kinase domain, and then efficiently transfers its phosphoryl group to the N-terminal receiver domain of OmpR at aspartate residue-55 through EnvZOmpR complex formation [24,25]. Upon phosphorylation, OmpR becomes an active dimer that exhibits enhanced DNA-binding ability specific for both the *ompC *and *ompF *genes. As the number of phosphorylated OmpR protein molecules increases, two events occur: OmpR binds not only to the high affinity binding sites upstream of the *ompF *promoter but also to the one low affinity-binding site. Binding to this low affinity site results in repression of *ompF *gene expression. So OmpF porin protein production is stopped. When OmpR binds to the three low affinity sites upstream of the OmpC promoters *ompC *gene expression is stimulated, more OmpC porin protein is made and appears in the outer membrane of the cell [26]. The summary of events is depicted in Figure 2.

## Events associated with low osmolarity

**Figure 3:**
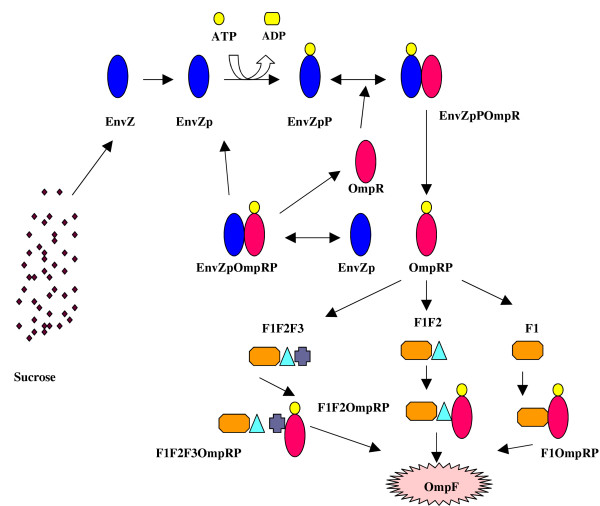
**Molecular Model of Osmoregulatory switch operative at Low Osmolarity in *Escherichia coli***. The low osmolarity conditions favour the phosphatase activity of EnvZ. The Phosphatase domain (EnvZp) upon autophosphorylation leads to formation of EnvZppOmpR complex and later favours the dephosphorylation of the cellular OmpRP thus making OmpRP available only for cooperative binding to the high affinity promoter of OmpF namely F1, F1F2, and F1F2F3 favouring OmpF expression.

In the low osmolarity state (Figure 3), however, EnvZ exhibits relatively low kinase activity (*i.e*., high phosphatase activity) towards OmpR. Such osmotic modulation of the kinase/phosphatase activity of EnvZ results in the relative amounts of the phosphorylated form of OmpR in cells varying in response to the medium osmolarity. When the medium osmolarity is low, the relative amount of the phosphorylated form of OmpR in cells is relatively small. In this particular situation, the *ompF *gene is first triggered, because the *ompF *promoter has relatively high-affinity OmpR-binding sites [27]. As the medium osmolarity increases, the relative amount of the phosphorylated form of OmpR increases proportionally, this in turn results in preferential activation of the *ompC *gene with low affinity OmpRP binding sites [9].

In summary, the relative amount of phosphorylated OmpR protein in the cell determines whether OmpF or OmpC is the predominantly expressed outer membrane porin. The relative amount of phosphorylated OmpR is determined by the perception of osmotic pressure by EnvZ [28].

## Results and discussion

### Simulation of low osmolarity

Reports have proposed that at low osmolarity, OmpRP binds cooperatively to F1, F1F2 and F1F2F3 sites resulting only in OmpF expression (Figure 3). Accordingly low osmolarity conditions were simulated assuming that *Escherichia coli *cells are grown in normal nutrient medium. The first event of start of simulation is the activation of phosphatase activity of EnvZ followed by autophosphorylation of EnvZp by ATP dissociation. Later EnvZpp-OmpR complex formation occurs (Figure 3) [29]. This short lived complex triggers phosphorylated regulator OmpRP. The phosphatase activity carries out the dephosphorylation of the phosphorylated regulator. Thus the concentration of cellular OmpRP is available only for binding of *ompF *promoters leading to OmpF porin expression.

**Figure 4:**
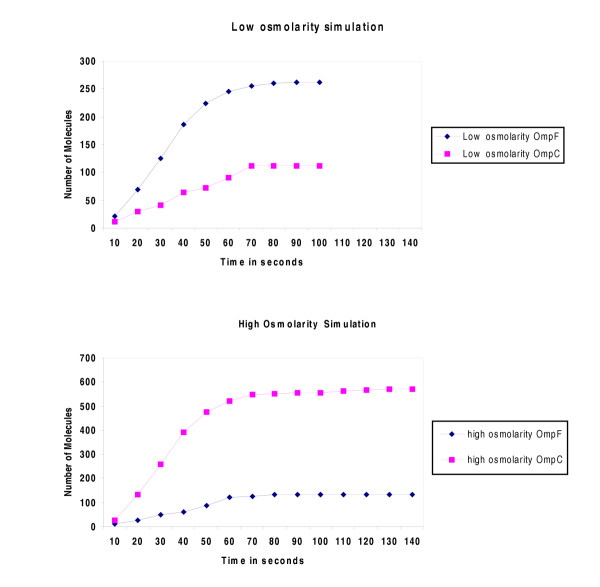
**Porin production at Low and High osmolarity conditions **Indicated in the X-axis is the time in seconds and number of molecules of the component in the osmoregulatory switch in Y-axis. (a) The start of simulation with OmpF (indicated in blue) synthesis gradually triggered. By mid term of simulation steady state production and final saturation of OmpF molecules could be seen. Shown in b is the OmpC (indicated in pink) production and reaching saturation levels at high osmolarity conditions.

In the case of Low osmolarity steady state production of OmpF and expression of OmpC could be seen initially at the start of simulation. However at the saturation of OmpF, repression of OmpC was significant. The steady state production of OmpF expression and OmpC repression is indicated in Figure 4a.

**Figure 5:**
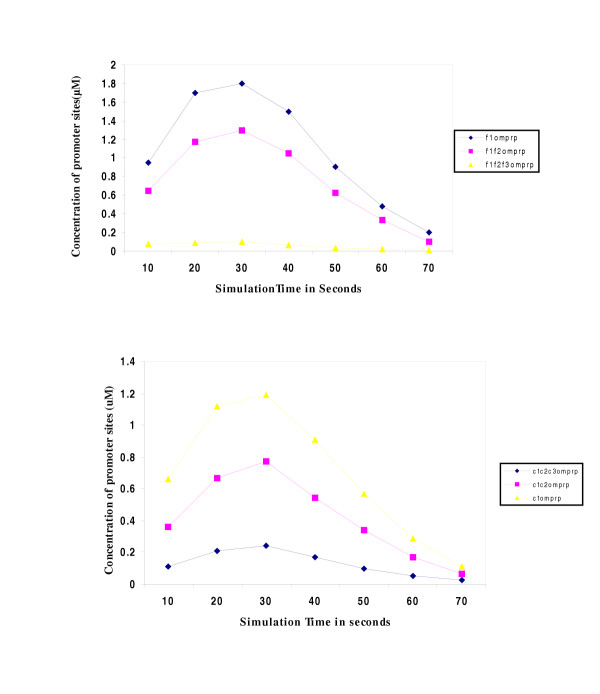
**Regulator-promoter complex formation simulation **The intermediate products of simulation namely the regulator-promoter complexes for regulation of (a) OmpF: f1omprp – blue; f1f2omprp – pink; f1f2f3omprp – yellow and (b) OmpC: c1omprp-yellow; c1c2omprp-pink; c1c2c3omprp-blue respectively.

The sucrose levels were maintained as normal (around 150 molecules). OmpF synthesis seen to be triggered at the start of simulation. The entire trend of OmpF synthesis, gradual increase, steady state (at 50 seconds after the start of simulation with about a constant increase of 10 molecules for every 10 seconds) and final saturation (near 90 seconds with an constant value of 262 molecules) are represented graphically. (Figure 4a). A similar trend is seen with OmpC molecules reaching saturation at 70 seconds. The intermediates of low osmolarity pathway namely promoter binding is indicated in Figure 5a. It could be seen that EnvZpOmpR complex is formed at very basal levels thereby directly having control over OmpC molecules. Additionally only 3.5% of cellular OmpR was phosphorylated at minimal sucrose in low osmolarity conditions validating the model that is operative in low osmolarity.

### Simulation of high osmolarity

For high osmolarity conditions, the model generated incorporates the concentration of sucrose with the assumption that *Escherichia coli *cells are grown in nutrient broth with 20% additional concentration of sucrose (1.11 M equivalent) [30]. With this injection stimulus, the kinase activity of EnvZ is enhanced leading to OmpC expression.

The first event of start of simulation is the activation of kinase activity of EnvZ followed by autophosphorylation of EnvZk by ATP dissociation. Shortly thereafter, EnvZkOmpR complex level rises and the dissociation of the complex raises the level of the phosphorylated regulator OmpR. This is followed by cooperative binding of OmpR to the *ompF *promoters and *ompC *promoters. OmpC molecules start accumulating. In the course of time there is complete dissociation of EnvZkOmpR complex, thereby both sensor and response protein are brought back to the pool (Figure 2). In our model sucrose levels turns the EnvZ to take up kinase activity by increasing EnvZk concentration [31]. In this model we show that the steady state production of OmpF is dramatically reduced in the high osmolarity medium. As the cooperative binding of OmpRP to *ompC *promoter sites is known to require a higher concentration of OmpRP than *ompF *promoter sites, this is achieved by reducing the EnvZ phosphatase activity. In this manner, a few fold increase in the OmpRP concentration on the cell is enough to induce OmpC expression and concomitant repression of OmpF expression by OmpRP binding to the F4 repressor site. Recent *in vivo *studies reveal that F4 site is key factor responsible for OmpF repression [32,28]. The rate of OmpC production increased notably after sucrose addition, which is comparable with literature results. OmpC expression reaching saturation and subsequent OmpF repression is indicated in Figure 4b.

In high osmolarity, the increase of sucrose level (to about 1.11 M equivalent) [30]*in silico *through the virtual pipette directly favours OmpC production. OmpC production follows the same trend as OmpF in steady state (from 60 seconds to 90 seconds with a constant increase of 13 molecules per second) and saturation reaches by 100 seconds. OmpF repression shows steady state increase of 6 molecules for every 10 seconds and finally at saturation takes up a constant value by 90 seconds (Figure 4b) but still the events are delayed compared to low osmolarity. Here again the key marker EnvZk is found in significant levels as seen in the graphical representation and so are the promoter-regulator complex (Figure 5b). Here 10% of cellular OmpR is finally phosphorylated at the end of simulation agreeing with the literature data.

### Effect of Volume changes over porin production

Shrinkage of *Escherichia coli *is associated with osmotic change [33]. The effect of volume increase and decrease over the porin production was verified with the simulation model. A volume decrease of 10% and 20% from the specified 10^-15 ^Litres was incorporated into the simulation model at low and high osmolarity conditions. In both cases the decrease in volume did not affect the relative porin production. Although the reach of saturation was rapid, the levels of porin and their relative ratios were found to be maintained with the same trend. This agrees with the data by Wood [34] with regard to the phases of the osmotic stress response by *Escherichia coli *K-12. The simulation model presented is at the first phase of physiological and structural responses triggered by osmotic shift where there is decreased cytoplasmic streaming. Our simulation time also corresponds with the time period of this first phase of the responses in the above literature. Table 2 represents the OmpC and OmpF levels at decreased volume levels.

### Effect of ATP changes over porin production

To address the issue whether ATP levels has any effect over relative porin levels, simulation run was done at high and low levels of ATP. ATP level of 3 and 5 mM has been reported in exponentially growing *Escherichia coli *cells [35,36]. Simulation was carried out at these two concentrations of ATP. Table 2 indicates relative porin levels at high and low ATP levels. As has been reported earlier, the ATP increase leads to plasmolysis, thereby leading to crowding of molecules [34]. This does not seem to affect the ratio of porins in the simulated system even though the reach of simulation was rapid. This is possibly due to the robust nature of the system.

### Porin production in the complete absence of the Sensor, EnvZ

Regulation of OmpC and OmpF expression in *Escherichia coli *in the absence of sensor, EnvZ has been studied [17]. We have thus examined the steady state production of OmpF and OmpC in the absence of EnvZ and also looked at the rate of production of the porins during osmolytic shift. As reported *in vitro*, EnvZ is required for the maximal OmpC production and for efficient induction of OmpC at high osmolarity. This is established in the *in silico *model. Also the lack of EnvZ in the simulation did not affect the OmpF at low osmolyte condition and incomplete OmpF repression could be noticed after osmolyte shift as reported by *in vitro *studies. The relative levels of porins without EnvZ is cross verified with the data reported by Frost *et al. *[17], over steady state production of OmpF and OmpC with minimal sucrose and osmolytic shift conditions (Table 2).

### Effect of Elevated EnvZ levels over porin production

Within the context of the EnvZ/OmpR two component system, the mathematical model predicts the OmpF/OmpC transcription to be insensitive to variations in the level of EnvZ and OmpR. By increasing levels of EnvZ upto 10 fold (1000 molecules), the relative porin ratio was found to be constant during simulation, evidently agreeing with the robust nature of the switch as per the mathematical model [35].

### Effect of OmpR levels over porin production

As with the case of EnvZ, through *in silico *model the OmpC/OmpF transcription was found to be independent of the OmpR levels upto 10-fold increase (20000 molecules). In either case both at high and low osmolarity, porins levels were found to be insensitive to elevated or decrease levels of response regulator, OmpR corresponding to the results of mathematical model (Table 2).

## Conclusion

Signaling pathways, for example, commonly operate close to points of instability, frequently employing feedback and oscillatory reaction networks that are sensitive to the operation of small number of molecules [37,38]. The model simulated here is clearly a simplified description of the EnvZ/OmpR system. There are a number of aspects of the circuit that have not been included such as EnvZ dimerization, conformational changes of OmpR [6] or additional enzymatic steps. The simulation is based on the mathematical model of the EnvZ-mediated cycle of phosphorylation and dephosphorylation [39]. Thus, this model predicts that the regulation of OmpF and OmpC as a direct consequence of the level of OmpR-P in the cell and is dependent on the way in which OmpR-P interacts with sites in the *ompF *and *ompC *regulatory regions [40]. Previously, it was suggested on the basis of a simplified model for the EnvZ-mediated cycle of phosphorylation and dephosphorylation of OmpR that the output of the circuit (the concentration of OmpR-P) should be independent of the concentration of EnvZ and OmpR in the cell [41]. We have shown porin regulation at high and low osmolyte concentrations where the dual activity of EnvZ is primarily controlled by the concentration of osmolyte stimulus at the start of simulation. The preliminary simulation experiment indicates that both reaching steady state expression and saturation is delayed in the case of OmpC compared to OmpF. The relative porin production seems to be unaltered with changes in cell volume, ATP, EnvZ and OmpR at low and high osmolarity conditions. But the reach of saturation was rapid at high and low osmolarity with altered levels of the above components. Experimental analysis will help improve the model. The model captures the basic features of the generally accepted view of EnvZ-OmpR signaling and is a reasonable starting point for building sophisticated models and explaining quantitative features of the system. At the same time, beyond its applicability to EnvZ-OmpR the model provides an interesting mechanism for achieving robust behavior with a bi-functional enzyme that may be broadly applicable to the other regulatory circuits within cells.

## Methods

**Figure 6:**
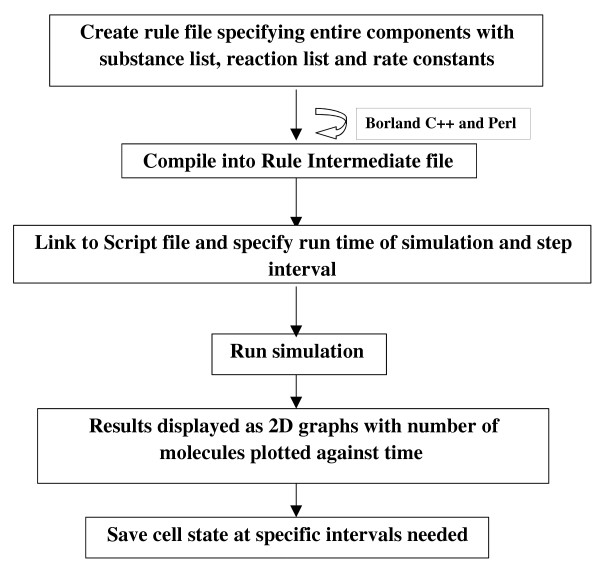
Schematic representation of steps in creation of simulation model using E-CELL system

The E-CELL Windows version 2.25 was employed for simulation [42,43]. The software was installed with the third party software namely Active Perl, JRE (java runtime environment) and Borland C++ compiler [44] essential for running simulations. The information defining all the components of the osmoregulatory switch, reactions and appropriate reactors and rate constants and environmental parameters describing volume was incorporated in the rule file. This file was further compiled through Active perl. The order of reaction kinetics, time of simulation and time interval was specified in script file. The reactor is basically a file describing /defining the kinetics of the equation along with rate constants. Reactor file were complied using C++ compiler. Figure 6 summarizes the method of construction of quantitative model.

### Creation of rule file based on the mathematical model

The computational model of osmoregulatory switch is based on the mathematical model by Goulian and Batchelor [39]. The entire model is described in the rule file. The cell system and cell environment was defined first. Changes in volume could not be incorporated, hence in this *in silico *approach, volume parameters were assumed constant. Also simulations with other system have assumed volume as a constant parameter irrespective of the system simulated. Simulations could not be defined and shown visually for volume parameter, as E-CELL has no provision for spatial information. Table 3 details the list of substances in osmoregulatory with their respective substance IDs.

### Reactor Specifications

E-Cell is based on an object-oriented modeling theory, structured Substance-Reactor Model (SRM). The simulation models are constructed with three fundamental object classes, Substance, Reactor and System. Substances represent state variables, Reactors represent operations on the state variables, and Systems represent logical and/or physical compartments containing other objects. The distributed package of version employed for carrying out simulation has 18 different classes of standard Reactors, such as for Michaelis-Menten formula and generalized chemical equilibrium [43]. In the simulation systems, the rate equations of all the reactions are defined. Every reaction follows different kinetics based on the substrate involved and is dependent on reaction type. The reactors employed for osmoregulatory switch includes mass action, Catalysed mass action (specified in Table 4) defining all reactions from sucrose injection till the regulated expression of the porins. Reactor is the term employed here, as in E-CELL, to describe the reaction rate. The volume of the system is assumed to be unchanged during simulation using a constant parameter reactor. Molecular binding such as osmolyte interaction and response regulator DNA binding was modeled using Mass Action reactor, which computes velocity as a product of concentration of substrates and a kinetic constant. The expression of porin was modeled on mass action principles with catalyst embedded using a Catalyzed Mass Action reactor. Autophosphorylation of sensor, sensor-regulator complex formation and ATP dissociation was modeled using MichaelisUniUni reactor. Auto dephosphorylation reactions was modeled using MichaelisUniUnireactor. Zeroreactor, which calculates velocity independent of concentration of molecular species, was employed for modeling complex dissociation. A Decay reactor was employed for defining the disintegration or decay of components. Table 4 details the reaction type and reactors with their respective chemical constants employed for constructing the model.

The present model is built on the assumption of the *in vivo *condition considering *Escherichia coli *cells grown in mid-log phase. Accordingly the levels of OmpR and EnvZ are reported to be 3500 and 100 molecules in cell respectively. OmpR and EnvZ levels were almost the same from cells grown in L-broth medium or in a high osmolarity medium (NB (Nutrient Broth) +20% sucrose) [26]. The ratio of OmpR to EnvZ is reported to be constant, assuming the cell volume to be 10^-15 ^liters [45]. At low osmolarity the phosphorylation of only 3.5%(120 nM or 70 OmpRP molecules/cell) of total OmpR molecules in a cell (2024 molecules OmpR molecules per cell) would be enough to activate the expression of OmpF, whereas at high osmolarity the phosphorylation of about 10%(590 nM or 350 OmpRP molecules /cell) of total OmpR molecules in a cell (3500 molecules per cell) would be sufficient to promote the expression of OmpC and to repress the expression of OmpF [46](Table 1). The majority of OmpR still remains unphosphorylated, as it's pool is very large. It is important to note that the osmoregulation of the OmpF and OmpC gene is finely tuned by having a very large pool of OmpR molecules [30]. The list of key substances participating in osmoregulatory switch with their initial concentration at the start of simulation is summarized in Table 5. The data was adapted from Cai and Inouye [30]. As the data with regard to the number of promoters was not available, different values were taken and checked with the porin production and their relative ratios. The relative ratios were found be unaltered with any promoter levels.

## Authors contribution

KVS was responsible for data collection and analysis. SK conceived of the study, and participated in its design and analysis. Two referees and an advisor of the journal helped to bring this information into the biological context.

## Figures and Tables

**Table 1:** Sensor and response regulator molecules at high and low osmolarity. Based on the assumed *in vivo *data as reported by Cai and Inouye, 2002 [30], the levels of the components EnvZ, OmpR and OmpRP at high and low osmolarity conditions are listed. At high osmolarity 10% of cellular OmpR gets phosphorylated, sufficient enough to promote OmpC expression. Contrarily at low osmolarity only 3.5% of cellular OmpRP would be sufficient enough to activate the expression of OmpF.

**SUBSTANCE**	**High osmolarity (no. of molecules/cell)**	**Low osmolarity (no. of molecules/cell)**
EnvZ	100	60
OmpR	3500	2100
OmpRP	350(10%)	70(3.5%)

**Table 2:** Effect of ATP, EnvZ, OmpR and volume at high and low osmolarity conditions. The levels of low and high ATP taken were 3 mM and 5 mM respectively. The volume taken was 10% and 20% decrease with respect to 10^-15. ^At low EnvZ, the level was taken as nil and was elevated till 10 fold (1000 molecules with respect to 102 reported value). Similarly for OmpR a 10 fold increase and decrease from the reported value of 2100 was taken (20000 and 200 molecules respectively)

**Condition**	**Low Osmolarity**				**High Osmolarity**			
	
	Saturation time OmpF	Number of OmpF	Number of OmpC	Ratio OmpF/OmpC	Saturation time OmpF	Number of OmpC	Number of OmpF	Ratio OmpC/Omp F
Low ATP	30	244	100	2.4	18	557	129	4.3
High ATP	26	242	106	2.2	13	557	130	4.2
Low EnvZ	120	246	6	41	85	550	246	2.2
High EnvZ	135	244	104	2.3	80	550	125	4.4
Low OmpR	125	35	13	2.6	70	52	14	3.7
High OmpR	130	2176	889	2.4	115	5309	1210	4.3
Low Volume	150	245	108	2.2	73	537	124	4.3
High Volume	130	243	100	2.4	80	548	125	4.3

**Table 3:** List of components involved in osmoregulatory switch

**Substance**	**Substance Description**	**Substance_ID**
Sucrose	Sucrose	s
EnvZ	Sensor kinase EnvZ	envz
EnvZk/EnvZp	EnvZ with Kinase / phosphatase activity	envzk/envzk
EnvZkP/EnvZpP	Phosphorylated EnvZ with Kinase / phosphatase activity	envzkp/envzpp
OmpR/OmpRP	Response regulatory OmpR/Phosphorylated OmpR	ompr/omprp
EnvZpPOmpR EnvZkPOmpR EnvZpOmpRP EnvZkOmpRP	Sensor kinase-regulatory complexes	envzppompr envzkpompr envzpomprp envzkomprp
F1 F1F2 F1F2F3 F1F2F3F4	*ompF *gene promoter sites	f1 f1f2 f1f2f3 f1f2f3f4
C1 C1C2 C1C2C3	*ompC *gene promoter sites	c1 c1c2 c1c2c3
F1OmpRP F1F2 OmpRP F1F2F3 OmpRP F1F2F3F4 OmpRP C1 OmpRP C1C2 OmpRP C1C2C3 OmpRP	Response regulator-promoter complex	f1omprp f1f2omprp f1f2f3omprp f1f2f3f4 omprp c1omprp c1c2omprp c1c2c3omprp
ATP	ATP	ATP
OmpF	Porin OmpF	ompf
OmpC	Porin OmpC	ompc

**Table 4:** Rate equations, reactors and respective rate constants employed in simulation. Represented in the table are the details of the reaction and reactor type along with kinetic constants. Theoretical and Kinetic data used for defining the rate equations taken from literature are quoted. Those indicated in * are defined as concentration/second.

Reaction	Equation	Constant	Comments
Formation of EnvZk and EnvZp	v = k [envz] [s] v = k [envz] [s]	k = 15.6 μM k = 0.15 μM	(High osmolarity) (low osmolarity) Catalysed Mass action reactor, velocity is calculated as a product of concentrations of substrates and kinetic constants [Data as presented by Yoshida et al [29]]
Formation of EnvZkp and EnvZpp	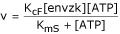 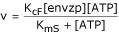	KmS = 1 μM* KcF = 10 μM* KmS = 10 μM* KcF = 100 μM*	As per chemotaxis data. Kinetics of the reaction described by Henri Michaelis Menten equation derived from rapid equilibrium assumptions [(Bray et al [4]]
Formation of EnvZkpompr and EnvZppompr complex	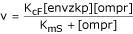 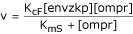	KmS = 0.51 μM* KcF = 10 μM* KmS = 0.42 μM* KcF = 20 μM*	As per chemotaxis data Kinetics of the reaction described by Henri Michaelis Menten equation derived from rapid equilibrium assumptions. [(Bray et al [4]]
Dissociation of EnvZkpompr, EnvZppompr, EnvZkomprp and EnvZpomprp complexes	v = rate	Rate = 1.20 μM	Represented by zero reactor, velocity is independent of concentration of molecular species [data as presented by Yoshida et al [29]]
Formation of F1omprp, F1F2omprp, F1F2F3omprp	v = k [f1] v = k [f1f2] v = k [f1f2f3] v = k [f1f2f3f4]	k = 6.8 nM k = 10.7 nM k = 15.4 nM k = 21.2 nM	Mass action reactor, velocity is calculated as a product of concentrations of substrates and kinetic constants [data as presented Head et al [28]]
Formation of C1omprp, C1C2omprp, C1C2C3omprp	v = k [c1] v = k [c1c2] v = k [c1c2c3]	k = 7.7 μM k = 18.9 μM k = 31.4 μM	Mass action reactor, velocity is calculated as a product of concentrations of substrates and kinetic constants [data as presented Head et al [28]]
Degradation of F1F2F3F4 OmpRp (OmpF repression)	[f1f2f3f4omprp]		In the reactor class decay process, substrate reduced according to the half-life inputted. [Bergstrom et al [28]]
Formation of OmpC and OmpF	v(OmpC) = k [c1c2c3omprp] [envzk] v(OmpF) = k [f1f2f3fomprp] [envzp]	k = 1 μM k = 1 μM	Catalysed Mass action reactor, velocity is calculated as a product of concentrations of substrates and kinetic constants (Batchelor and Goulian [39])

**Table 5:** Initial levels of molecular species at the start of simulation. Indicated in the table are initial concentrations of substances at the start of simulation for low and high osmolarity conditions. At high molarity the sucrose molecules added (virtually through substance window) to the medium is indicated in the table. EnvZ-Sensor kinase, OmpR-Response regulator [data as presented by Cai and Inouye] [30], F1, F1F2, F1F2F3-*ompF *gene promoter sites and C1, C1C2, C1C2C3-*ompC *gene promoter sites. Envzp, envzk, envzppompr, envzkpompr, envzpomprp, envzkomprp, f1omprp, f1f2omprp, f1f2f3omprp, f1f2f3f4omprp, c1omprp, c1c2omprp, c1c2c3omprp are initially nil at the start of simulation. During the course of simulation, these complexes are formed and dissociated at the end of simulation

**Species**	**Number of molecules**	
	
	**High**	**Low**
**EnvZ**	113	102
**OmpR**	3500	2100
**Sucrose**	150	3000
**f1**	-	100
**f1f2**	-	100
**f1f2f3**	-	100
**c1**	100	-
**c1c2**	100	-
**c1c2c3**	100	-
**ATP**	100	100
